# A roadmap to clinical trials for FLASH

**DOI:** 10.1002/mp.15623

**Published:** 2022-04-25

**Authors:** Paige A. Taylor, Jean M. Moran, David A. Jaffray, Jeffrey C. Buchsbaum

**Affiliations:** ^1^ The University of Texas MD Anderson Cancer Center Houston Texas USA; ^2^ Memorial Sloan Kettering Cancer Center Manhattan New York USA; ^3^ Radiation Research Program Division of Cancer Treatment and Diagnosis National Cancer Institute National Institutes of Health Bethesda Maryland USA

**Keywords:** advanced technology, clinical trials, FLASH, quality assurance, radiation therapy, ultra‐high dose rate

## Abstract

While FLASH radiation therapy is inspiring enthusiasm to transform the field, it is neither new nor well understood with respect to the radiobiological mechanisms. As FLASH clinical trials are designed, it will be important to ensure we can deliver dose consistently and safely to every patient. Much like hyperthermia and proton therapy, FLASH is a promising new technology that will be complex to implement in the clinic and similarly will require customized credentialing for multi‐institutional clinical trials. There is no doubt that FLASH seems promising, but many technologies that we take for granted in conventional radiation oncology, such as rigorous dosimetry, 3D treatment planning, volumetric image guidance, or motion management, may play a major role in defining how to use, or whether to use, FLASH radiotherapy. Given the extended time frame for patients to experience late effects, we recommend moving deliberately but cautiously forward toward clinical trials. In this paper, we review the state of quality assurance and safety systems in FLASH, identify critical pre‐clinical data points that need to be defined, and suggest how lessons learned from previous technological advancements will help us close the gaps and build a successful path to evidence‐driven FLASH implementation.

## 1 INTRODUCTION

The current FLASH radiation therapy paradigm is defined as dose delivered to a treatment volume at over approximately 50 Gy per second. These dose rates are believed to cause less damage to normal tissue than at standard therapy dose rates (2‐10 Gy per minute) while also maintaining the tumoricidal effect. While there are efforts to investigate this in the pre‐clinical setting,^1^, ^2^, ^3^ state‐of‐the‐art clinical trials will be the critical step in evaluating the efficacy of FLASH. In this paper we illuminate the critical quality assurance components that must be addressed for safe and reproducible clinical trials of FLASH radiotherapy.

Some historical data suggested indirectly that dose rate can significantly impact radiation biology. Perhaps the first publication describing a dose rate effect was by Sax who in 1939 noted that when radiation at an “ultra‐high dose rate” of 600 R/min was compared to 2 R/min, chromosomal (double strand) breaks increased with the dose rate but total (single strand) breaks were not changed significantly.^4^ A 1958 paper by Kirby‐Smith and Dolphin confirmed that “two‐hit aberrations were only 40%–50% of the frequency seen at a dose rate of 1 × 10^6^ rads per second when the rate was increased to 4 × 10^8^ rads per second in air,” hinting at the current FLASH observations.^5^ Interestingly, when the same cell culture experiment was performed in nitrogen, no difference was seen in two‐hit aberrations. In 1971 Hornsey and Bewley showed that very high dose rate electron therapy caused local hypoxia in gut and hypothesized that this caused relative radiation resistance which in normal tissue could be seen as protection.^6^ Recent publications have been unable to support oxygen depletion as the basis for what is being called the FLASH effect at current, much higher dose rates (over ∼50 Gy/s), and at present, the etiology for the current FLASH effect is unknown.^7^, ^8^

The scientific organizations at the heart of radiation oncology, such as AAPM, ASTRO, ESTRO, the NCI, and NRG Oncology are actively working toward addressing many technical issues and providing guidance so that clinical exploration of FLASH can proceed. The commercial sector is supporting clinical trials with FLASH‐capable proton systems and developing treatment planning software dedicated to optimizing FLASH delivery. However, major technology gaps still exist for how to develop robust, conformal, and inversely‐optimized patient plans and no vendor, to our knowledge, has developed a real time dose monitoring system that captures enough information to interrupt or safely resume a FLASH treatment. Ironically, the one open trial in the US at the time of the drafting of the paper is using pre‐planned and pre‐validated treatment plans, much as was done in the distant past in photon radiotherapy.

Given that proton therapy is still being assessed in randomized clinical trials, the early experience of protons offers a cautionary tale as FLASH trials are developed. Several proton therapy clinical trials did not show an overwhelming advantage of proton therapy.^9^, ^10^ Non‐superiority of proton therapy in early trials was explained to be a result of still‐developing technology (i.e., the results would improve once treatment planning and beam delivery technology improved), but some studies also highlighted safety concerns with the increased radiobiological effect of proton therapy on normal tissue.^11^, ^12^, ^13^ Much research has been done to account for this radiobiological effect and reduce the risk of proton radionecrosis,^14^, ^15^ but the results of these early studies put a tremendous burden on the ongoing proton trials to show overwhelming survival and toxicity benefits. Insurance companies, meanwhile, have used early studies as justification not to pay for proton therapy, making clinical trial enrollment more challenging. FLASH trials should take these challenges to heart when designing protocols. The more rigorously we test early phase trials, the more success we can expect later with larger, multi‐institutional trials.

### 1.1 Establishing standards for FLASH dosimetry and addressing quality assurance and safety gaps

The infrastructure that exists for normal quality assurance (QA) in radiation oncology is extensive and necessary given the capacity of radiation therapy machines to do serious harm if there is a malfunction or significant error in any part of the treatment planning and delivery process. Examples of rigor in the medical physics space include AAPM Task Group 100,^16^ which reviewed risk in the delivery of IMRT, and proton therapy robustness of plan reporting data elements standardization.^17^ Part of this rigor is the requirement that dose in normal (non‐FLASH) radiation oncology be traceable to national and international standards. The major patient safety gaps for FLASH, or ultra‐high dose rate (UHDR), radiation are with respect to calibration methodologies and techniques, and the need for active onboard dosimetric monitoring which could be used to interrupt incorrect treatment deliveries.

### 1.2 Calibration and machine quality assurance considerations

With respect to safety, the importance of calibration procedures for absolute dosimetry cannot be overstated. Consistent calibration methodologies are needed for all FLASH delivery techniques. The reproducibility of dose rate is essential. Such work will be crucial for clinical trials, as will the impact of FLASH on treatment planning^18^, ^19^, ^20^, ^21^ and intensity modulated/VMAT delivery.^22^, ^23^ If technical gaps remain as clinical trials are developed, we can leverage the experience with IMRT and begin with clinical trials in treatment sites that are more tolerant to simple beam geometries and easy to localize. More complex sites can be pursued as the robustness of commercial systems is improved to support sophisticated treatment planning and more complex delivery with image guidance and motion management.

One of the biggest challenges is achieving accurate and precise dosimetry at the elevated dose rates used in FLASH. There are no commercially available dosimetry devices for UHDR at this time, so we are unable to accurately and independently measure the dose compared to that reported by the manufacturer of the FLASH delivery device. A number of publications examine limitations of ion chamber dosimetry in a UHDR beam.^24^, ^25^, ^26^ The AAPM, ESTRO, the IAEA and other organizations have a long history of providing guidance for new technologies through reports.^27^, ^28^, ^29^ AAPM report TG 359, which is being jointly developed with AAPM, ESTRO and EFOMP, will provide guidance on dosimetric equipment and methods for calibrating the dosimetry of FLASH systems. The aims for the TG are to 1) review dosimetry uncertainty and the need for standardization in UHDR experiments, 2) assess radiation measurement equipment for UHDR, and 3) provide guidelines for calibration, dosimetry, and reporting. The TG will not be developing a calibration protocol, which will still need to be addressed.

While the AAPM and IAEA have calibration protocols for photons, electrons, protons, and heavy ions, the protocols are not designed for ultra‐high dose rates.^30^, ^31^, ^32^ A number of factors would need to be determined to adapt these protocols for UHDR, including ion recombination in reference ion chambers, appropriate field size for calibration (many FLASH‐capable machines can only deliver field sizes smaller than the common 10 cm × 10 cm reference field), beam quality (e.g., accounting for the changing energy spectra caused when the machines are modified), and traceability to a primary standard. A European consortium of industry, clinical, and research partners, UHDpulse—Metrology, has been formed to develop metrology and codes of practice for UHDR irradiations in electron and proton beams.^33^ Broader global engagement, including by the US, should be encouraged to assure harmonization of protocol development efforts.

### 1.3 During treatment beam monitoring—dose and positioning considerations

Beyond calibration, beam monitoring is extremely important in the context of UHDR and high dose deliveries. From a machine delivery perspective, errors and issues have been observed with output stability for machines,^34^ systematic calibration errors,^35^ and interlock failures^36^ with potentially severe safety risks. If some machines struggle with stability at conventional dose rates and lower dose levels, how can we trust they will be stable at high dose rates and total doses? It's important for machine reproducibility to be tested, and for interlocks to be in place to prevent radiation delivery errors. Proper characterization of monitor chamber dose response, including for the very brief exposure times as described above, will be crucial for intra‐fraction monitoring, interlock activation, and recovery.

Interrupting a FLASH treatment quickly enough to avoid a misadministration is clearly a non‐trivial task and no publicly available testing data exist showing a commercial system is able to stop delivery of a FLASH dose quickly enough to address this need. The safety concerns are serious, given historical radiation accidents like the AECL Therac‐25 error that resulted in fatal over‐irradiations of patients.^37^ This issue is indirectly acknowledged in the paper by the Swiss group who treated the first human with FLASH using electrons with the following statement: “In contrast to a standard monitor chamber installed on a clinical radiotherapy LINAC, the system was not coupled to a beam stopping device that could interrupt the beam in case the beam properties were out of tolerance.^38^” If the planned treatment is not being delivered in desired FLASH time structure, even a rapidly stopped plan might give large areas of normal tissue too much dose causing potentially significant side effects. For this reason, FLASH planning needs to not only be optimized for time/dose rate issues but also consider interruptability and safety. It is also important to be able to safely resume treatment should the treatment target remain in the correct position if the delivery is interrupted. This is currently not the case. As treatment planning systems are developed, it will be necessary to develop strategies that are robust to the types of motion or errors that may occur. An example of using planning to support robust delivery was seen for some IMRT delivery systems, which used a feathering technique between two split fields for those optimized fluence maps which required a carriage move to deliver the full treatment field. Such approaches will need to be re‐thought in the context of FLASH due to the dependence on both dose and dose rate to achieve the desired therapeutic ratio.

Little has been published on IGRT for UHDR delivery. While conventional IGRT techniques may prove appropriate for addressing inter‐fraction motion for a UHDR setup, timing tolerances for intra‐fraction motion could be quite tight due to the sub‐second total irradiation time of a UHDR irradiation. Alternatively, the potential for reduced toxicity with UHDR could be used to relax the need for tighter PTV/ITV margins provided dose is not escalated. Given the single beam restriction on FLASH delivery for the foreseeable future, they are likely to rely on 3D volumetric localization followed by real‐time image‐based monitoring that triggers the brief UHDR exposure. One could imagine MR‐based systems being integrated for their improved tissue contrast and real‐time monitoring capability, but such developments are in the distant future.

In addition to patient positioning, the positioning of beam shaping and modulating devices needs to be carefully verified. For example, several proton manufacturers are experimenting with non‐uniform ridge filters to achieve modulation over a target area. Due to the nonuniformity of these devices, they are highly sensitive to positioning, and small errors in alignment can cause significant changes in the dose distribution and local dose rates. It may be necessary to perform patient QA with the field filter immediately prior to treatment delivery. Photon FLASH raises its own interesting questions. Intensity modulation would likely have to move toward patient‐specific device manufacturing given the impossibility of MLC movement in a reproducible fashion in a FLASH time domain. As noted, treatment planning methods will need to be developed to support the design of new ancillary devices and employ more robust planning techniques.

### 1.4 Clinical trial QA

In the clinical trial context, external peer review of programs and delivery systems will be an important step to verify accuracy of dose delivery, and consistency between institutions on multi‐center trials. At a minimum, we feel it is critical to have the dosimetry of each system checked independently with vendor‐agnostic equipment similar to the standard we hold other medical radiation devices. If it is determined that a minimum dose rate is required to observe the FLASH effect, achieving this threshold will need to be tested as well. The clinical trial QA offices, such as the Imaging and Radiation Oncology Core (IROC) in the US, should develop tools to verify the absolute dose, dose rate, and timing of delivery. As mentioned previously, many tools like passive dosimeters and phantoms could be developed for these purposes, but first need to be verified. For example, IROC does not currently have a phantom designed for superficial lesions commonly treated in FLASH experiments, and while many dosimetric audit tools are dose rate independent (e.g., TLD, OSLD, film, etc.) at conventional dose rate, most have yet to be verified in UHDR beams. Time should be provided to develop, test, and disseminate guidance for a rigorous testing process to validate dosimeters for dose measurements in UHDR beams. The Global Harmonisation Group,^39^ a partnership of international clinical trial QA centers and clinical trial groups, should be engaged to help develop consensus guidelines on other clinical trial QA for FLASH, such as minimal technology capabilities for dose and patient monitoring, patient case review, and delivery robustness analysis.

**FIGURE 1:**
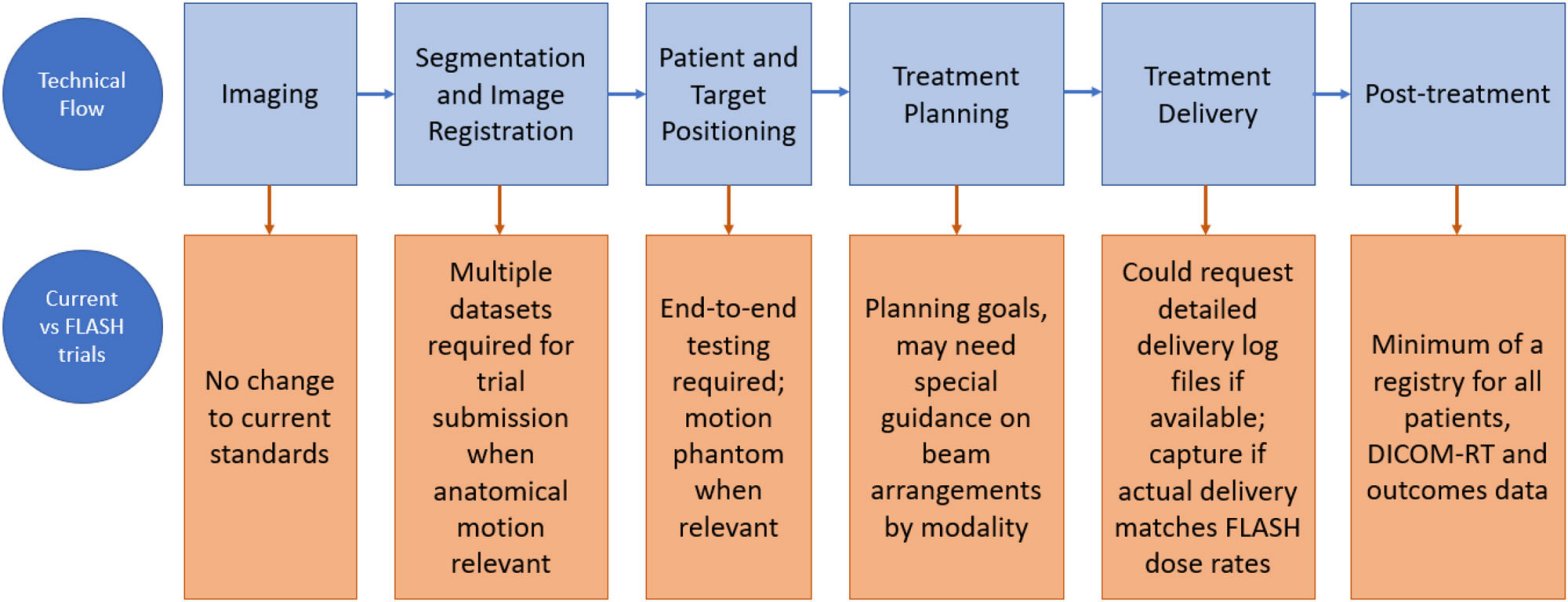
For each of the technical components of FLASH RT, we provide guidance on whether standard RT tools and workflow are sufficient or new technology and processes need to be developed. For trials during the early stages of UHDR use, it may be prudent to expand credentialing activities and to capture time‐related data. (Adapted from AAPM Task Group 113 on Physics practice standards for (conventional) clinical trials).^57^

Figure 1 shows some of the considerations which may adversely impact clinical trials using FLASH techniques if not properly accounted for when compared to clinical trials for conventional radiation therapy.

### 1.5 Critical questions for reproducibility in clinical trials

With various methodologies and technologies to deliver FLASH radiation it becomes critical to define steps to reproduce treatment benefit from the FLASH effect.^40^, ^41^, ^42^ Four key questions that arise in defining FLASH therapy in clinical trials are presented below.

First, how well can we select, measure, optimize, and reproduce the fine structure of the UHDR dose delivery process.^43^ While this is likely to be a work in progress, the first to be developed would need to be the capacity to measure FLASH in real‐time to provide for the needed safety systems like we have on standard dose‐rate devices today. In other words, can we safely fully define, optimize, deliver, and store a FLASH plan's delivery history in a standard fashion so that all needed parameters are recorded for later review? For this we will need planning capacity, measurement capacity, and further extensions made to the DICOM‐RT standard.

Second, can we measure if the biological response to FLASH treatment varies from patient to patient and from tumor to tumor from day to day, in particular if medications or drugs vary the critical biology of FLASH therapy? The large fractions potentially needed for FLASH make this more critical because if the FLASH effect is not present for a particular patient for some reason, that patient is likely being treated with a larger fraction, or perhaps with less dose shaping, than they would otherwise have received with standard techniques. To prevent this potentially catastrophic error, it would be useful to assess whether and where a given patient is having the biological “FLASH effect,” ideally in real time and necessarily in only definitive treatment scenarios as palliative scenarios neither fully test safety nor efficacy. In a field where we do not accept a 5% severe toxicity rate, will this limitation be tenable based on our inability to measure biology fast enough to achieve damage avoidance for all patients?

Third, can FLASH treatment be delivered across realistic, deep and/or larger volumes. To date, FLASH data has been studied in relatively small target volumes. If protective of normal tissue, we want FLASH to be used in all areas of normal tissue, while avoiding this same sparing in tumor tissue. For spot scanning protons, the speed a spot can be scanned across an area will prevent uniformity of FLASH dose rate. Will that result in complex patterns of variable FLASH effect/elevated damage? How will that affect long term tissue damage? We similarly do not know if FLASH effects happen across multiple fields in the same fraction if the total time is not sufficiently fast for FLASH dose rates to be achieved overall. It is not clear that FLASH will be useful in curative settings if sharp dose gradients are required for this reason.^44^, ^45^, ^46^ For example, is there a FLASH penumbra on the edge and beyond the Bragg peak where dose delivery falls away from the FLASH regime possibly causing increased harm? Older treatment methods such as three‐dimensional conformal photon therapy or passive scanning proton therapy may offer more robust solutions for FLASH delivery than newer methods.

Fourth, how easy will it be to introduce FLASH therapy into the overall care matrix of a patient? Do other aspects of clinical care impact FLASH and do side effect management strategies for regular radiation therapy work with FLASH? Biological factors that can inadvertently be changed during the course of a clinical trial such as via diet or other transient exposures (heat, sleep, hydration, acidosis, baseline medications in use, transient illness, prior therapy including prior radiation, comorbidities, etc.) may alter or even abrogate the FLASH effect. Beyond just the binary issue of it works or does not work of the second issue, what if FLASH can be made to work but the variables cannot be modulated well so that it is partially effective? To study FLASH may require more sophisticated data collection to ensure control of other variables. For example, presently we require treatment once per day on trials for the most part. A FLASH trial may require a specific time of day, specific diet, or a finite number of medications.

Other papers in this issue highlight the variety of radiotherapy types used to deliver UHDR to date: electrons, photons, protons, and light ions.^47^, ^48^, ^49^ Multi‐modality clinical trials will require careful consideration of machine capabilities, differences in dose prescriptions, radiobiology, etc. Early clinical trials would optimally be powered to see if different FLASH modalities caused different outcomes using patients as their own controls if possible.

## 2 DISCUSSION

**FIGURE 2:**
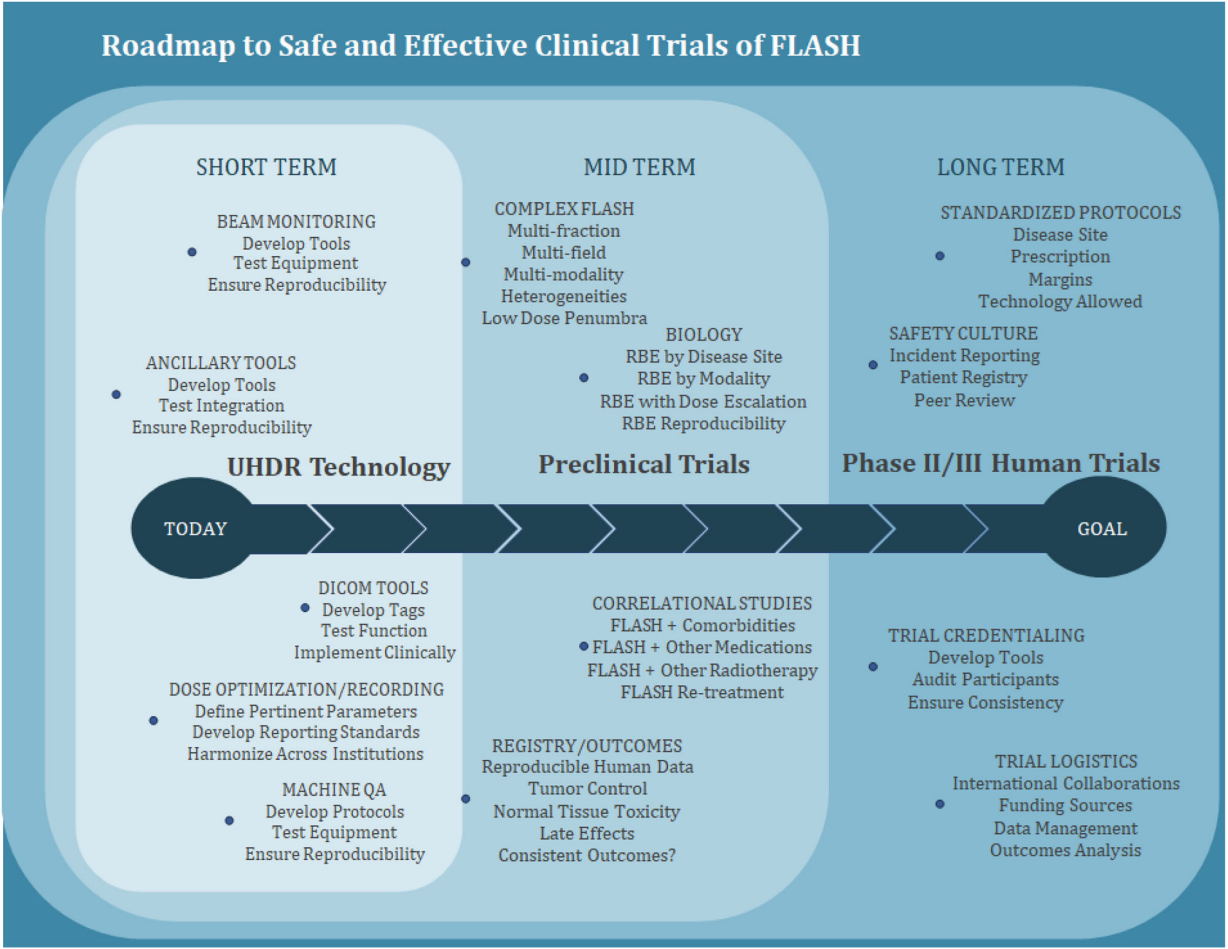
A roadmap highlighting key components that will be needed to successfully conduct Phase II/III clinical trials in humans. Many of the technological issues are being actively addressed, while preclinical trials are still being organized and standardized. This is contingent on reasonable understanding of the tumor and normal tissue biology within the study

Once safety efficacy data are gathered, things will get even more complex. How do we compare FLASH to the very safe standard of care with relatively few side effects at baseline? For example, if we decide it is time to do a large trial comparing FLASH to standard of care, it is a challenge to envision doing a comparison of FLASH whole brain. Would the clinical trial investigators be willing at multiple fractions of over 6 Gy to the whole brain in humans and or to allow a control to be done that is not fractionated. Thus, the best of standard of care may not be so much worse than FLASH even if FLASH proves to work. Possibly FLASH could even be better (or more toxic) once chemotherapy is added. Figure 2 outlines a consideration of a roadmap to multi‐institutional trials.

### 2.1 Recommendations

**FIGURE 3:**
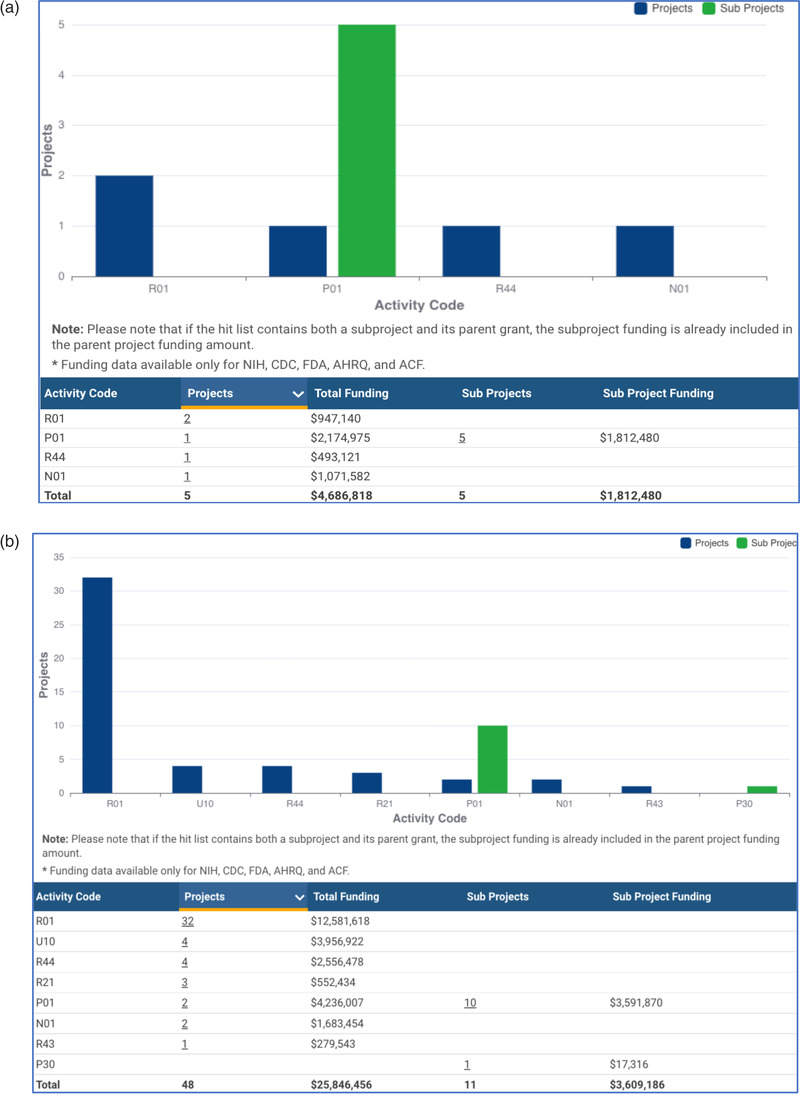
NCI annual funding support of FLASH related research can be viewed by the public on the website reporter.nih.gov.^58^ Shown are two screen captures from that website. Capture “a” is dated in June 2021 and “b” from January 2022. Both are from a search on keywords “FLASH” and “radiation therapy.” We defer to the reader to explore other global funding agencies’ data as publicly available

1. We suggest that organizations such as AAPM, ESTRO, and EFOMP continue to collaborate on guidance. While guidance is developed, the organizations may want to incorporate pilots to provide rapid feedback to refine their appropriateness and completeness. In certain areas, it may be advantageous for professional organizations to create a compendium of guidance on this emerging technology to support consistency in the process from simulation through treatment delivery and ultimately the monitoring of patient outcomes. It will also be essential to have robust manufacturer specifications that are designed based on the needed accuracy for patient treatments. International Electrotechnical Commission (IEC) standards will need to be updated to address FLASH‐capable systems.^50^
2. Since the time structure is such an important component of UHDR delivery, it will be important to collect the time structure information in clinical trials to ensure equipoise and retrospective analysis. While dose rate (MU/min or particles/min) can be saved in DICOM format, they are optional fields, nominal, and rarely versus time. Institutions would need to ensure these data were saved and submitted. Other delivery timing components, like instantaneous dose rate, pulse rate, and delivery time are not typically recorded in DICOM, but it would be important to collect those data as well. Some research has been done on log file validation of FLASH beam delivery,^51^ but log files are not typically collected as part of clinical trial data submission. The DICOM committee will need to make these reporting tools available and establish standards for the essential fields of the input (such as DICOM‐RT plan, structure, and image data) as well as a standard DICOM file of the delivery to confirm use of FLASH dose rates. We recommend the clinical trial groups build tools that can be used across trial groups and across treatment planning platforms to save the necessary treatment delivery data.
3. The determination of whether technology is considered equivalent to existing technology or new by regulatory authorities tasked with safety within the healthcare space is a decision by those authorities (e.g., the United States Food and Drug Administration). Because of the significant change in the physical devices, validation tools, software to control the devices, the FDA may end up considering these to be new devices. We recommend that clinical trials under development be constructed to allow for this possibility to avoid necessary regulatory delays.
4. As an emerging technology, incident reporting through systems such as ASTRO AAPM Radiation Oncology Incident Learning System (RO‐ILS), ROSIS, SAFRON, and other systems will require collaboration to ensure that any events that are identified at one institution can potentially be mitigated at other institutions. It may be beneficial to use a common incident learning system to capture near misses or events (with or without harm) that may be involved in the care for all patients treated with UHDR. Early clinical trials might consider rapid publication, presentation and access to ongoing clinical data given the newness of this technology.
5. Late effects happen late.^52^ Even if we overcome the technical issues noted above and develop a proper, achievable definition of parameters to achieve FLASH biological effect in real time, the reason FLASH is of interest is that it might decrease normal toxicity and broaden the therapeutic window. We do not have the decades of experience we have with standard dose rates so caution is required. We recommend that trials be properly powered and resourced to study late effects.
6. Clinical equity of access is important globally. Hyperthermia can offer a lesson: it was a promising methodology that was known to work but which was very difficult to deliver in practice due to many skill‐based reasons.^50^, ^51^ It's possible only some centers will be able to deliver FLASH. Global access to radiation therapy is far from ideal presently. We should try to design clinical trials in FLASH so as not to further exacerbate these issues. Studying methods that can be adapted broadly should be prioritized if practical to do so.
7. When conducting FLASH clinical trials, we recommend that general standard of care radiobiology research be studied alongside FLASH radiobiology much like normal tissue should be studied with tumor tissue when exploring ways to improve the therapeutic ratio. A significant proportion of current FLASH research involves particle therapy. We do not fully understand the relative biological effect (RBE) of protons and heavier ions and we don't fully understand standard photon radiobiology. We should be leveraging all the new ‐omic and data science (AI/ML/imaging) tools we have currently to advance both. Figure 3 shows current funding in this space at the NCI.

NCI (United States) recognizes these issues and currently has mechanisms that allow for competitive grant submission to study FLASH radiation from bench to clinical trials. Additionally, and just to use NCI as an example again, two related funding opportunities to study, (1) high LET radiation biology^55^ and (2) the radiobiology of the standard of care,^56^ have been recently put forward. These two programs represent about $7 million dollars per year in direct funding support. Data from these programs could have a positive impact on FLASH studies. Other global funding agencies also fund these areas of research.

## 3 CONCLUSIONS

FLASH radiotherapy is an exciting and important area of research that holds potentially great promise. It might allow a significant broadening of the therapeutic index for the field of radiation therapy and in doing so, allow more patients to be cured and have fewer side effects, but we need to walk before we run with FLASH trials. The bedrock of FLASH clinical trials will be the knowledge and infrastructure for FLASH QA so we can perform high quality, safe clinical trials to answer the burning questions of radiobiology and enable personalized cancer treatments. If we do not do trials correctly with FLASH the first time, we might not get another chance.

## FUNDING

National Institute of Health, Blue Cross Blue Shield of Michigan, and Varian Medical Systems; National Institute of Health grant CA180803.

## DISCLAIMER

This article represents the opinion of the authors. It does not represent the opinion or policy of the National Institutes of Health of the US Federal Government.

## CONFLICT OF INTEREST

Dr. Buchsbaum reports none.

Dr. Jaffray reports royalties from Beaumont Hospital/Elekta/Varian (RT cone‐beam CT), University Health Network/Elekta (SRS/SRT cone‐beam CT), University Health Network/Raysearch (deformation modeling), University Health Network/Modus (QA phantoms), and University Health Network/Precision X‐ray (small animal irradiator). He holds 26 US and 21 foreign patents (12 are/were licensed) and has another 8 inventions active in the patent process in the domain of radiation oncology and cancer treatment. He currently has no active research agreements and no consulting arrangements with industry.

Dr. Moran reports funding within the past year from the National Institute of Health, Blue Cross Blue Shield of Michigan, and Varian Medical Systems. Dr. Moran is part of a patent application related to dosimetry measurements (PCT/US2020/032385).

Ms. Taylor reports funding from the National Institute of Health grant CA180803.
